# A novel nomogram for predicting liver metastasis in patients with gastrointestinal stromal tumor: a SEER-based study

**DOI:** 10.1186/s12893-020-00969-4

**Published:** 2020-11-25

**Authors:** Guowei Zhou, Keshuai Xiao, Guanwen Gong, Jiabao Wu, Ya Zhang, Xinxin Liu, Zhiwei Jiang, Chaoqun Ma

**Affiliations:** 1 Department of General Surgery, Jiangsu Province Hospital of Chinese Medicine, Affiliated Hospital of Nanjing University of Chinese Medicine, Nanjing, 210029 Jiangsu Province China; 2 Department of General Surgery, Xinyang Central Hospital, Xin Yang, 464000 Henan Province China; 3 Department of Pediatrics, Jiangsu Province Hospital of Chinese Medicine, Affiliated Hospital of Nanjing University of Chinese Medicine, Nanjing, 210029 Jiangsu Province China; 4 Department of Gynecology, Jiangsu Province Hospital of Chinese Medicine, Affiliated Hospital of Nanjing University of Chinese Medicine, Nanjing, 210029 Jiangsu Province China

**Keywords:** Gastrointestinal stromal tumors, Liver metastasis, Nomogram, SEER

## Abstract

**Background:**

Liver metastasis (LIM) of gastrointestinal stromal tumor (GIST) is associated with poor prognosis. The present study aimed at developing and validating nomogram to predict LIM in patients with GIST, thus helping clinical diagnosis and treatment.

**Methods:**

The data of GIST patients derived from Surveillance, Epidemiology, and End Results (SEER) database from 2010 to 2016, which were then screened by univariate and multivariate logistic regression for the construction of LIM nomogram. The model discrimination of LIM nomogram was evaluated by concordance index (C-index) and calibration plots, while the predictive accuracy and clinical values were measured by decision curve analysis (DCA) and clinical impact plot. Furthermore, we validated predictive nomogram in the internal testing set.

**Results:**

A total of 3797 patients were enrolled and divided randomly into training and validating groups in a 3-to-1 ratio. After logistic regression, the significant variables were sex, tumor location, tumor size, N stage and mitotic rate. The calibration curves showed the perfect agreement between nomogram predictions and actual observations, while the DCA and clinical impact plot showed the clinical utility of LIM nomogram. C-index of the nomogram was 0.812. What’s more, receiver operating characteristic curves (ROC) also showed good discrimination and calibration in the training set (AUC = 0.794, 95% CI 0.778–0.808) and the testing set (AUC = 0.775, 95% CI 0.748–0.802).

**Conclusion:**

The nomogram for patients with GIST can effectively predict the individualized risk of liver metastasis and provide insightful information to clinicians to optimize therapeutic regimens.

## Background

Gastrointestinal stromal tumor (GIST) is a rare neoplasm of the gastrointestinal (GI) tract, which is considered to originate from the multipotential mesenchymal stem cells and differentiate to interstitial Cajal’s cells (ICC) [1–3]. The incidence of GIST has increased in the recent years, possibly related to the rapid development of endoscopic technology [1]. GIST can occur anywhere throughout the GI tract, stomach and small intestine are the most common site, followed by colon, rectum, esophagus [4, 5]. However, there were several studies indicated that GISTs can also arise outside of the GI tract, including pancreas, gallbladder, liver, retroperitoneum and so on [4, 6, 7].

It was reported that more than half of the GIST patients had metastases upon presentation at medical institutions, and the liver was the most common site [8]. Although complete surgical resection with negative margins is the standard treatment for GIST, over 50% of patients develop recurrence or metastasis [9, 10], with liver metastasis being the main metastatic pattern of GIST [11, 12]. Hence, the poor prognosis of GISTs might be related to the status of liver metastasis (LIM). Prior to the introduction of adjuvant therapy, the treatment of metastatic GIST was limited and the outcomes were dismal [13]. Tyrosine kinase inhibitors (TKIs) like imatinib have revolutionized the management of metastatic GIST for the marked improvements in survival outcomes [14]. Nonetheless, secondary mutations and drug resistance appeared during the adjuvant TKIs treatment, indicating that it is difficult to obtain the complete cure by the use of TKIs [15, 16]. Hence, the tools for predicting the biological behavior and clinical outcome of GIST assumed a crucial role in the management of GIST [17].

In recent years, Memorial Sloan-Kettering Cancer Center (MSKCC) nomogram has been worldwide used to generate the probability of a clinical event through a complex computational formula [18, 19]. With the aid of nomogram, clinicians can assess the risk of the clinical event and then design individual treatment plans, determine the use of adjuvant therapy, optimize aspects of therapies and consider appropriate patient counselling [20]. Considering the important role of LIM in the prognosis of GIST, the study presented in evaluating patients with GIST and discovering patients with high-risk scores in liver metastasis by the use of nomogram.

## Methods

### Data source and inclusion criteria

Considering that Surveillance, Epidemiology, and End Results (SEER) database started to provide data regarding the specific sites of metastatic GIST since 2010 [21, 22], we extracted data about patients with known histological diagnosis of GIST between 2010 and 2016 by the use of the SEER*Stat software version 8.3.6. The International Classification of Diseases for Oncology, 3rd edition (ICD-O-3) morphology codes (8936/3) were used to identify GIST.

**Fig. 1:**
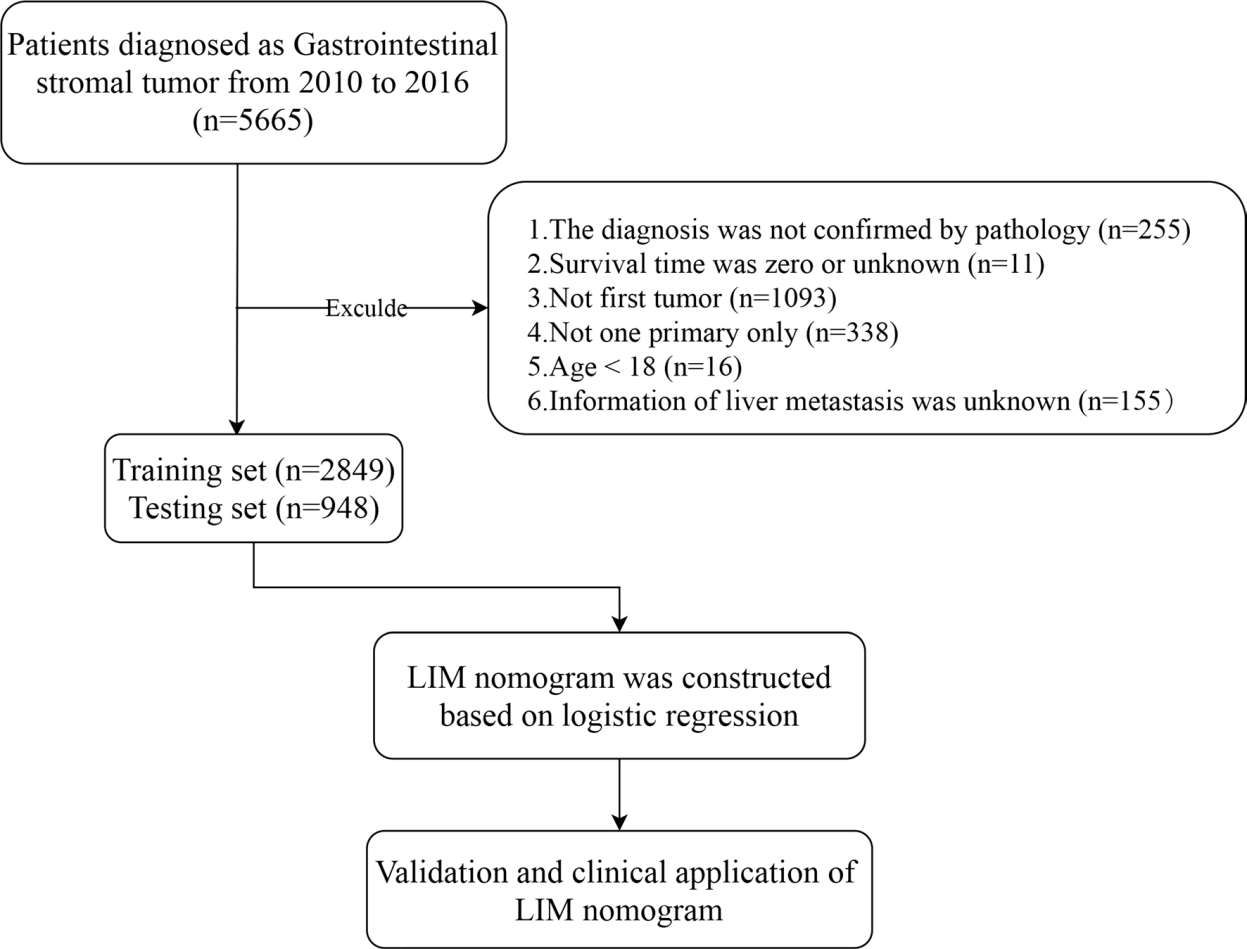
Study flowchart

The patient's inclusion and elimination process are shown in Fig. 1. We excluded the cases if they: (1) had no positive pathology; (2) had unknown survival time; (3) had not been the first tumor; (4) had more than one primary tumor; (5) age < 18 [23, 24]; (6) had unknown liver metastasis information.

We extracted the data from the SEER database including age, sex, race, marriage, primary site, N stage, tumor size, and mitotic rate, which was identified by the variable “CS site-specific factor 6”. The N stage was established according to the American Joint Committee on Cancer (AJCC) Cancer Staging Manual (7th edition). “SEER Combined Mets at DX-liver (2010+)” was used to identify the presence of liver metastasis in a newly diagnosed GIST patient. We classified patients as married, unmarried (including single, divorced, separated, and widowed) and unknown. Age was categorized into two groups: less than or equal to 65 years old and more than 65 years old [25]. Tumor locations with less than 20 cases were classified as “Others” [25].

### Construction, validation and clinical utility of nomogram

The GIST patients who met the inclusion criteria were randomly divided into training and testing sets in a 3-to-1 ratio. Afterward, the following variables were selected into the research: age, sex, marriage, race, tumor location, tumor size, mitotic rate and N stage. Univariate and multivariate binary logistic regressions were then used to identify the significant characteristics with the help of the forwarding stepwise selection method [26]. By the use of Hosmer–Lemeshow test, bootstrapping with 1,000 samples were utilized to internal validation of the nomogram and to draw calibration plots, which can indicate the association among the apparent curve, bias-corrected curve and ideal curve [27]. Harrell’s concordance index (C-index) and the receiver operating characteristic (ROC) were also employed to estimate the predictive performance of the nomogram. The higher C-index and the area under ROC curve (AUC) were, the better discrimination ability or prognostic accuracy the variables would be. Meanwhile, sensitivity and specificity of the cutoff values were then obtained [28]. Furthermore, the decision curve analysis (DCA), which plots net benefit (NB) at a range of reasonable risk thresholds that conform with the clinical practice, was applied to evaluate the clinical utility of nomograms for decision making [29]. Based on DCA, we developed clinical impact plots to visually show the estimated high-risk patients' number for each risk threshold [30].

### Statistical methodologies and software

Continuous variables were presented as mean values ± standard deviation (SD), and categorical variables were summarized as proportions. Continuous variables and categorical variables were compared by the Student’s *t*-test and Chi-squared test, respectively. IBM SPSS Statistics, version 26.0 (SPSS Inc, Chicago, IL, USA) and R software version 3.6.2 (http://www.r-project.org) performed the statistical methods mentioned above, and several R packages, including regplot, rms, rmda and pROC were applied to draw graphs, such as nomogram, calibration plot, DCA plot, and ROC curve. All P values were two-sided with values of P < 0.05 were considered statistically significant, and confidence intervals (CIs) stated at the 95% confidence level.

## Results

### Demographic baseline characteristics

3797 patients (Additional file 1: Table S1) fulfilled the inclusion criteria being enrolled and divided randomly into training and validating groups in a 3-to-1 ratio, with a training group (n = 2849) (Additional file 2: Table S2) for the construction of nomogram and a testing group (n = 948) (Additional file 3: Table S3) for internal verification, which was then summarized in Table 1. There was no statistically significant difference between training and testing sets (P > 0.05). LIM was present in 320 of 2849 patients (11.3%) in the training set, while the proportion that LIM occupied in the testing group was 11.5% (109 of 948). Afterward, as indicated in Table 2, in the correlation analysis, five clinical characteristics, including sex, tumor location, tumor size, N stage and mitotic rate were significantly correlated (P < 0.05) with LIM both in the training and testing groups.

**Table 1:** Baseline characteristics of patients

Characteristics	SEER cohort (%)			P
	Entire cohort n = 3797	Training n = 2849	Testing n = 948	
Age	62.25 ± 13.67	62.12 ± 13.90	62.38 ± 13.43	0.616
Sex				0.962
Male	1908(50.2)	1431(50.2)	477(47.1)	
Female	1889(49.8)	1418(49.8)	471(52.9)	
Marriage				0.158
Married	2143(56.4)	1609(56.4)	534(56.3)	
Unmarried	1447(38.1)	1096(38.4)	351(37.0)	
Unknown	207(5.5)	144(5.2)	6.6(6.7)	
Race				0.359
White	2534(66.7)	1909(67.0)	625(65.9)	
Black	684(18.0)	519(18.2)	165(17.4)	
Others	579(15.3)	421(14.8)	158(16.7)	
Tumor location				0.716
Stomach	1509(39.7)	1119(39.2)	390(41.1)	
Duodenum	213(5.6)	158(5.5)	55(5.8)	
Jejunum	272(7.1)	208(7.3)	64(6.7)	
Ileum	104(2.7)	86(3.0)	18(1.8)	
Colon	79(2.0)	60(2.1)	19(2.0)	
Rectum	104(2.7)	79(2.7)	25(2.6)	
Others	457(12.0)	340(11.9)	117(12.3)	
Unknown	1059(28.2)	799(28.3)	260(27.7)	
Tumor size				0.003
≤ 2 cm	446(11.7)	356(12.4)	90(9.4)	
2–5 cm	1066(28.0)	797(27.9)	269(28.3)	
5–10 cm	1088(28.6)	839(29.4)	249(26.2)	
> 10 cm	896(23.5)	645(22.6)	251(26.4)	
Unknown	301(8.2)	212(7.7)	89(9.7)	
N stage				0.319
N0	3531(93.1)	2658(93.4)	873(92.2)	
N1	131(3.4)	97(3.4)	34(3.5)	
Unknown	135(3.5)	94(3.2)	41(4.3)	
Mitotic rate				0.794
< 5/50 HPFs	1835(48.3)	1386(48.6)	449(47.3)	
≥ 5/50 HPFs	814(21.4)	610(21.4)	204(21.5)	
Unknown	1148(30.3)	853(30.0)	295(31.2)	
Liver metastasis				0.823
No	3368(88.7)	2529(88.7)	839(88.5)	
Yes	429(11.3)	320(11.3)	109(11.5)	

*SEER* Surveillance, Epidemiology, and End Results, *HPFs* high power fields

**Table 2:** Correlations between characteristics of patients with liver metastasis in the training and testing groups

Characteristics	Training set (%)			Testing set (%)		
	Negative	Positive	P	Negative	Positive	P
Age	62.18 ± 13.77	61.67 ± 14.86	0.560	62.41 ± 13.62	62.10 ± 11.96	0.802
Sex			< 0.001			0.004
Male	1238(48.9)	193(60.3)		408(48.6)	69(63.3)	
Female	1291(51.1)	127(39.7)		431(51.4)	40(36.7)	
Marriage			0.254			0.818
Married	1442(57.0)	167(52.1)		474(56.4)	60(55.0)	
Unmarried	960(37.9)	136(42.5)		308(36.7)	43(39.4)	
Unknown	127(5.1)	17(5.4)		57(6.9)	6(5.6)	
Race			0.144			< 0.001
White	1695(67.0)	214(66.8)		556(66.2)	69(63.4)	
Black	451(17.8)	68(21.2)		145(17.2)	20(18.3)	
Others	383(15.2)	38(12.0)		138(16.6)	20(18.3)	
Tumor location			< 0.001			< 0.001
Stomach	1026(40.5)	93(29.0)		357(42.5)	33(30.2)	
Duodenum	140(5.5)	18(5.6)		51(6.0)	4(3.6)	
Jejunum	186(7.3)	22(6.8)		59(7.0)	5(4.5)	
Ileum	83(3.2)	3(0.9)		16(2.1)	2(1.8)	
Colon	51(2.0)	9(2.8)		18(2.1)	1(0.9)	
Rectum	75(2.9)	4(1.2)		23(2.7)	2(1.8)	
Others	266(10.5)	74(23.1)		85(10.1)	32(29.1)	
Unknown	702(28.1)	97(30.6)		228(27.5)	32(29.1)	
Tumor size			< 0.001			< 0.001
≤ 2 cm	338(13.3)	18(5.6)		86(10.2)	4(3.6)	
2–5 cm	755(29.8)	42(13.1)		256(30.5)	13(11.9)	
5–10 cm	766(30.2)	73(22.8)		221(26.3)	28(25.6)	
> 10 cm	534(21.1)	111(34.6)		215(25.6)	36(33.3)	
Unknown	136(5.6)	76(23.9)		61(7.4)	28(25.6)	
N stage			< 0.001			< 0.001
N0	2412(95.3)	246(77.0)		791(94.2)	82(75.2)	
N1	60(2.4)	37(11.5)		28(3.3)	6(5.5)	
Unknown	57(2.3)	37(11.5)		20(2.5)	21(19.3)	
Mitotic rate			< 0.001			< 0.001
< 5/50HPFs	1329(52.5)	57(17.5)		428(51.0)	21(19.2)	
≥ 5/50HPFs	557(22.0)	53(16.5)		187(22.2)	17(15.5)	
Unknown	643(25.5)	210(66.0)		224(26.8)	71(65.3)	

*HPFs* high power fields

### Univariate and multivariate logistic regression results

In the univariate and multivariate logistic regression analyses, there were finally five parameters significantly correlated with LIM (Table 3), namely sex (female: odds ratio (OR) 0.723, 95% CI 0.577–0.939, P = 0.015), tumor location (rectum: 0.226, 0.079–0.647, P = 0.006), tumor size (> 10 cm: 2.842, 1.649–4.897, P < 0.001; unknown: 4.251, 2.359–7.660, P < 0.001), N stage (positive: 3.313, 2.057–5.337, P < 0.001; unknown: 2.851, 1.723–4.716, P < 0.001) and mitotic rate (≥ 5/50 HPFs: 1.803, 1.210–2.687, P = 0.004; unknown: 5.763, 4.091–8.116, P < 0.001).

**Table 3:** Risk factors for liver metastasis identified by univariate logistic regression analysis and multivariate logistic regression analysis

Characteristics	Univariate logistic regression analysis			Multivariate logistic regression analysis		
	OR	95% CI	P	OR	95% CI	P
Age			0.336			
≤ 65	1					
> 65	0.890	0.701–1.129	0.336			
Sex			< 0.001			0.015
Male	1			1		
Female	0.631	0.498–0.800		0.723	0.557–0.939	0.015
Race			0.146			
White	1					
Black	1.194	0.892–1.599	0.233			
Others	0.786	0.547–1.129	0.192			
Marriage			0.254			
Married	1					
Unmarried	1.223	0.962–1.556	0.101			
Unknown	1.156	0.680–1.965	0.593			
Tumor location			< 0.001			0.057
Stomach	1			1		
Duodenum	1.418	0.831–2.421	0.200	0.994	0.554–1.784	0.985
Jejunum	1.305	0.799–2.130	0.287	1.403	0.826–2.381	0.210
Ileum	0.399	0.124–1.286	0.124	0.377	0.110–1.293	0.121
Colon	1.947	0.929–4.079	0.078	0.686	0.312–1.511	0.350
Rectum	0.588	0.210–1.645	0.312	0.226	0.079–0.647	0.006
Others	3.069	2.198–4.285	< 0.001	1.043	0.708–1.537	0.831
Unknown	1.524	1.129–2.058	0.006	1.049	0.752–1.463	0.778
Tumor size			< 0.001			< 0.001
≤ 2 cm	1			1		
2–5 cm	1.045	0.593–1.841	0.880	1.123	0.623–2.024	0.700
5–10 cm	1.790	1.052–3.045	0.032	1.584	0.910–2.755	0.104
> 10 cm	3.903	2.329–6.541	< 0.001	2.842	1.649–4.897	< 0.001
Unknown	10.493	6.048–18.026	< 0.001	4.251	2.359–7.660	< 0.001
N stage			< 0.001			< 0.001
N0	1			1		
N1	6.046	3.932–9.296	< 0.001	3.313	2.057–5.337	< 0.001
Unknown	6.365	4.123–9.824	< 0.001	2.851	1.723–4.716	< 0.001
Mitotic rate			< 0.001			< 0.001
< 5/50 HPFs	1			1		
≥ 5/50 HPFs	2.219	1.507–3.266	< 0.001	1.803	1.210–2.687	0.004
Unknown	7.615	5.599–10.365	< 0.001	5.763	4.091–8.116	< 0.001

*OR* odds ratio, *95% CI* 95% confidence interval, *HPFs* high power fields

### Construction and validation of LIM nomogram

**Fig. 2:**
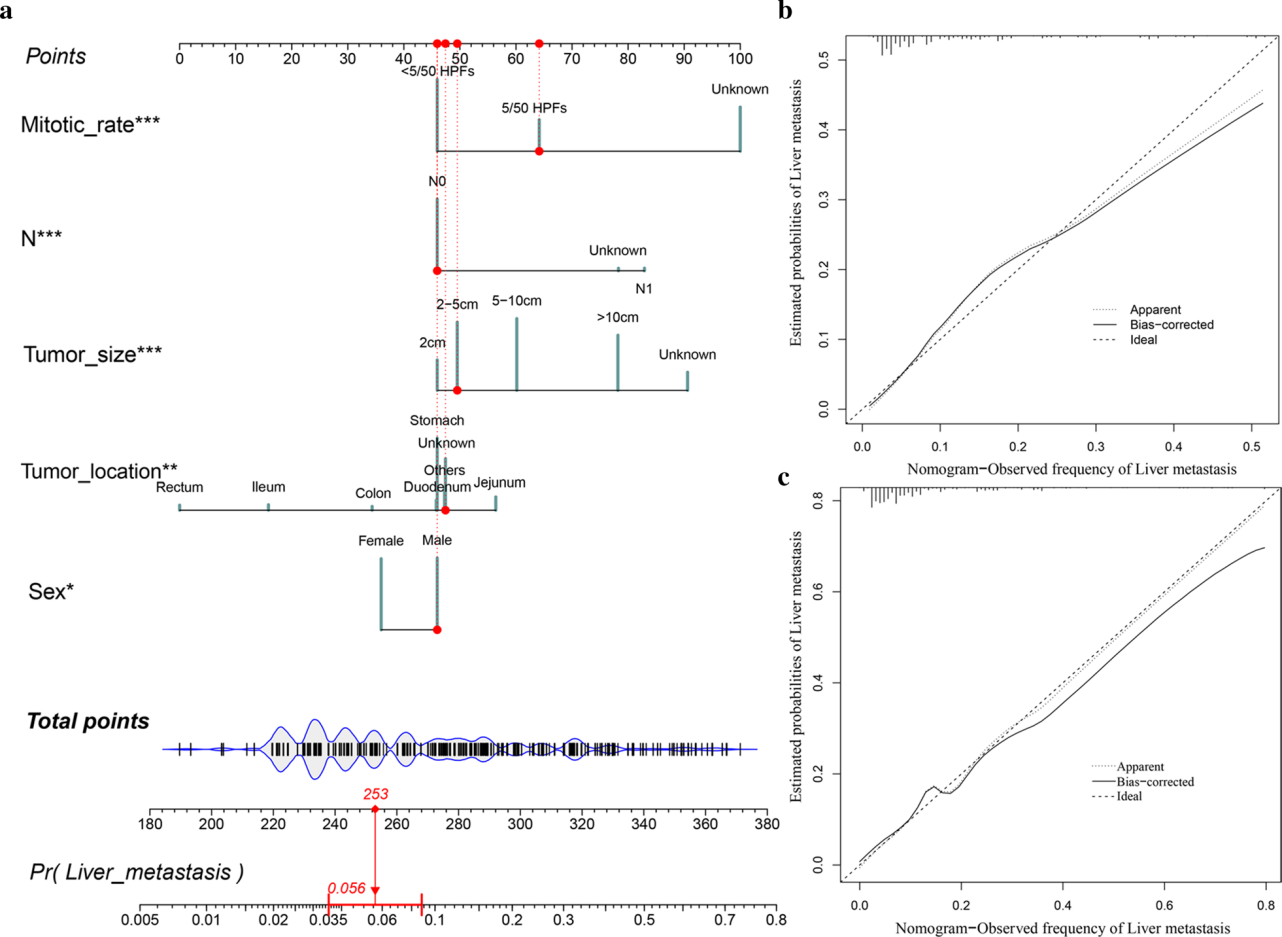
Nomogram and calibration curves for the prediction of liver metastasis in the patients with GIST. There are five characteristics enrolled in the LIM nomogram (**a**), and the patient #77784053 is illustrated by mapping its values to the covariate scales. Calibration curves for predicting LIM in the training groups (**b**) and testing groups (**c**) are shown in the right side (Bootstrap = 1000 repetitions). The detailed statistics are provided in Additional file 2: Table S2 and Additional file 3: Table S3. Abbreviations: *LIM* liver metastasis, *Pr* prediction

**Fig. 3:**
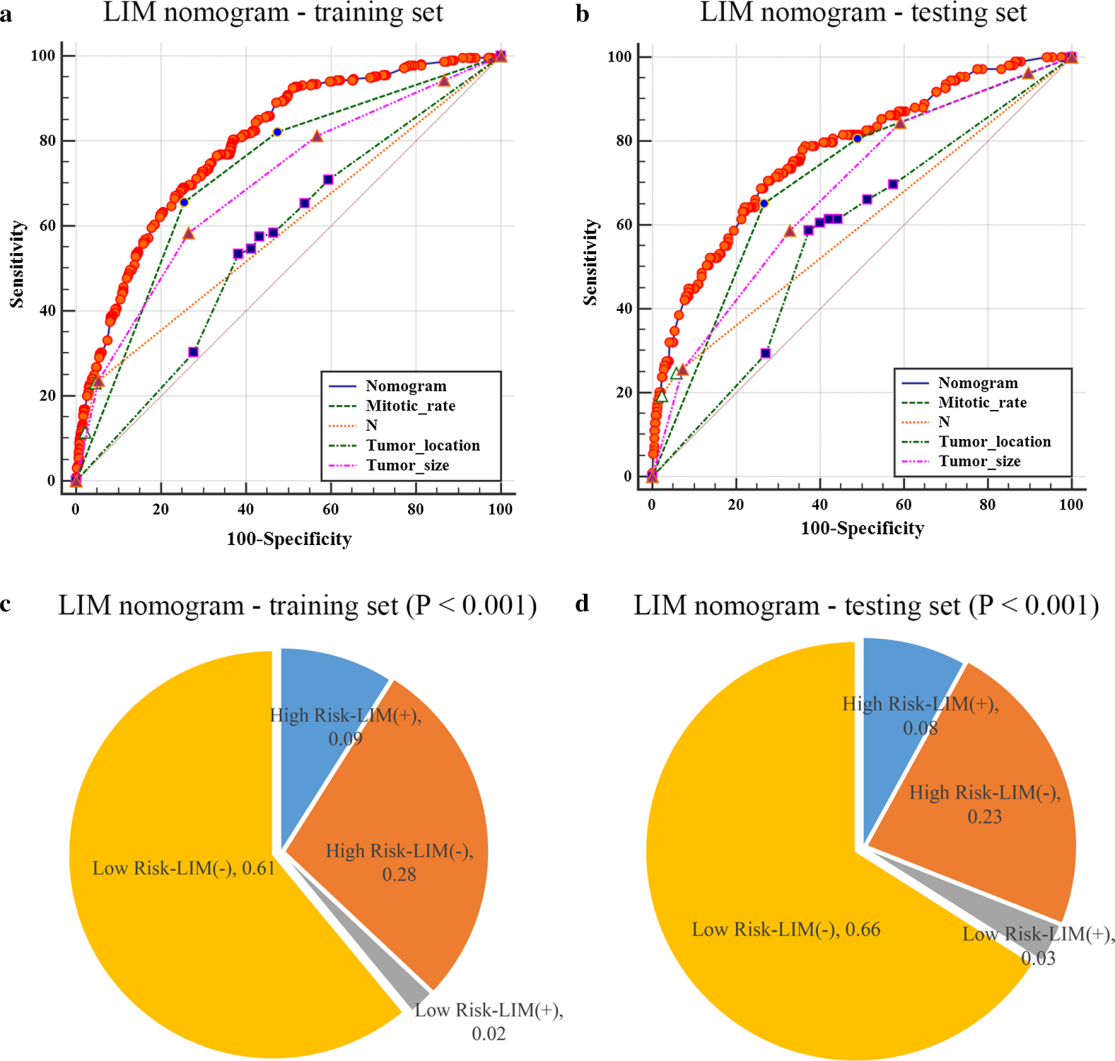
Receiver operating characteristic (ROC) curve analysis for LIM nomogram and pie charts for indicating the discriminatory power of LIM nomogram. In the training (**a**) and testing (**b**) groups of LIM nomogram, the AUC was respectively 0.794 (95% CI 0.778–0.808) and 0.775 (95% CI 0.748–0.802). The P values were two-sided. The maximum Youden index of the ROC curves were employed to distinguish the risk of liver metastasis in the training group (**c**) and the testing groups (**d**), respectively. The detailed statistics are provided in Additional file 2: Table S2 and Additional file 3: Table S3. The P values were two-sided and tested by Chi-square test. Abbreviations: *ROC* receiver operating characteristic, *LIM* liver metastasis, *95% CI* 95% confidence interval

The results of univariate and multivariate logistic regression were then used to construct LIM nomogram (Fig. 2a). As demonstrated in the LIM nomogram, mitotic rate was expectedly to be the best predictor, followed by the tumor size, tumor location, N stage and sex. Afterward, the calibration plots of the nomogram (Fig. 2b, c) indicated that apparent curve, bias-corrected curve, and ideal curve were well numerically agreed both in the training and testing groups. Respectively, the AUC values of the nomogram were 0.794 (95% CI 0.778–0.808) and 0.775 (95% CI 0.748–0.802) in the training and testing groups (Fig. 3a, b). According to the ROC curves in the training set, the value of nomogram was more significant than other variables, including tumor location (AUC 0.562, 95% CI 0.544–0.581, P < 0.001), mitotic rate (0.725, 0.708–0.741, P < 0.001), tumor size (0.697, 0.679–0.714, P < 0.001) and N stage (0.593, 0.574–0.611, P < 0.001). Similarly, in the testing set, the value of nomogram was also more significant than mitotic rate (0.711, 0.680–0.739, P < 0.001), tumor size (0.682, 0.652–0.712, P < 0.001), N stage (0.598, 0.566–0.629, P < 0.001) and tumor location (0.574, 0.542–0.606, P < 0.001). Furthermore, by the use of the maximum Youden index in the training group [19], the cutoff values of 170 and 188 were get, the patients were then divided into high-risk and low-risk groups with the results shown as pie charts (Fig. 3c, d).

### Clinical utility of LIM nomogram

**Fig. 4:**
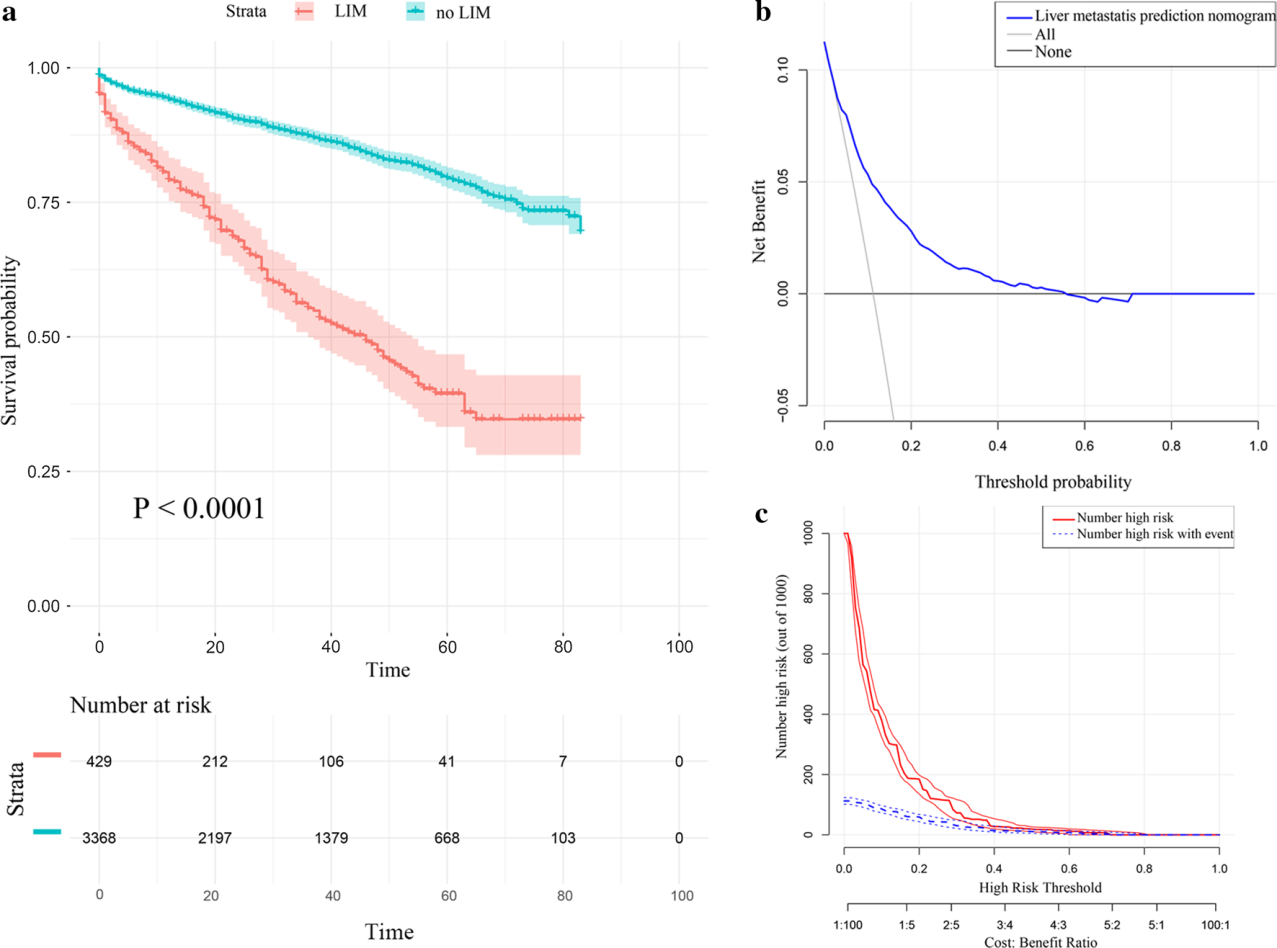
Kaplan–Meier survival curve, decision curve analysis, and clinical impact plot of patients with GIST. The entire cohort of patients with GIST were enrolled to construct the Kaplan–Meier survival curve (**a**). The decision curve analysis (DCA) and clinical impact of the LIM nomogram (**b**, **c**) in the training group are plotted. The detailed statistics are provided in Additional file 2: Table S2 and Additional file 3: Table S3. The P value was two-sided. Abbreviations: *LIM* liver metastasis

Firstly, we developed Kaplan–Meier survival curves of overall survival (OS) for all 3797 patients (Fig. 4a) enrolled in the study and there were significant differences between the Kaplan–Meier survival curves of the two sets (P < 0.001), which indicated that patients with GIST who were predicted to have LIM would have significant survival disadvantage. Afterward, as shown in DCA curve (Fig. 4b), threshold probabilities of 0.02–0.54 was the best benefit to LIM. Furthermore, clinical impact plot (Fig. 4c) of the training set indicated that, during the most beneficial threshold probabilities range, the predicted high-risk patients were always more than the patients actually had LIM, accompanying with acceptable cost–benefit ratios.

## Discussion

GIST was mistaken for schwannomas, leiomyomas or leiomyosarcomas until the introduction of ultrastructural, immunohistochemical, and molecular biological techniques, which uncovered that GIST originated from myenteric nervous system [31], and ICCs were further suggested to be the cells of origin [32]. Subsequently, gain-of-function mutations in the tyrosine kinase receptor KIT and platelet-derived growth factor receptor-α (PDGFRA) were groundbreakingly found as the main oncogenic driver in GIST [33, 34], which encouraged the development of GIST targeted therapies [5]. In recent researches, Etwenty-six (ETS) variant 1 (ETV1) was also reported to overexpress in GIST and enhance the expression of KIT when binding target genes [1, 35]. However, nearly 10–15% of adult GIST and 85% of pediatric GIST are negative for KIT and PDGFRA mutations, as called wild-type (WT) GIST, which is a component of the Carney-Stratakis syndrome caused by the succinyl dehydrogenase (SDH)-mutations [36]. Whereas, liver was the most common site that GIST metastatic to, both in WT GIST and non-WT GIST, and LIM of GIST was always suggested to be related to the poor prognosis [37].

Nomogram, a quantitative tool for assessing risk and benefit, has been widespread applied in the medical domain for clinical decision-making [38]. In the previous studies, several nomograms have been developed and validated to predict the OS, cancer-specific survival (CSS) of GIST, recurrence-free survival (RFS) and disease-free survival (DFS) after surgical resection of GIST [25, 39–41]. However, the nomogram for predicting LIM of GIST has not yet been reported.

In the present research, we ultimately obtained 3797 GIST cases from SEER database and the LIM nomogram was further established based on five significant patient features, namely sex, tumor location, tumor size, N stage and mitotic rate. The discrimination performance, as demonstrated in the calibration plots (Fig. 2b, c), was assessed by an internal bootstrap resampling approach. Furthermore, as shown in the ROC curves (Fig. 3a, b), the LIM nomogram displayed better diagnostic efficiencies when compared with the other single variables, and the AUCs of the nomogram were 0.794 (0.778–0.808) and 0.775 (0.748–0.802) in the training and testing groups, respectively.

Although the classification, line(s) of differentiation, prognostication have long been the confusion and controversy of GIST, tumor size and mitotic rate were the widely accepted risk factors. By means of tumor size and mitotic rate, the first risk classification of GIST, constructed by Fletcher et al. [42], divided GIST patients into four sets, including very low risk, low risk, intermediate risk, and high risk. Previous studies indicated that the size and mitotic rate of GIST were proportional to poor prognosis [25, 39, 40], which corroborated our results. However, metastatic measures were not uncommon for postoperative GISTs, even that with small tumor size and low mitotic rate [43]. The results obtained from the multivariate analysis of Yang et al.’s study [11] presented that GIST size was not a significant prognostic factor of the liver metastatic GIST. In contrast, Mietinenn et al. suggested tumor size was the metastatic risk of GIST, rather than mitotic rate [44]. In our opinions, GIST with larger tumor size or higher mitotic rate tend to subject to earlier adjuvant therapy and more extensive resections, which may be a reason for the discrepancy in the previous studies [11, 45, 46]. Additionally, male GIST patients were more likely to have LIM than female ones, which was in accordance with the previous studies that indicated GIST had a mild male predominance [11, 47], however, the underlying mechanism remains unclear.

Recently, controversy exists surrounding the relationship between primary tumor location and prognosis of GIST. It is a common dogma that gastric GIST (G-GIST) has a more favorable behavior when compared with small intestinal GIST (SI-GIST) [17]. Together with tumor size and mitotic rate, Miettinnen et al. added tumor location as a poor prognostic factor for the construction of Armed Forces Institute of Pathology (AFIP) classification [42, 48]. Based on approximately 2000 cases, SI-GIST resulted in a relatively higher risk of metastasis and tumor-related death, particularly with the tumor size exceeding 5 cm [48]. Anatomic site was also reported to be the significant independent predictor of OS [49], CSS [50] and RFS [51], with SI-GIST accompanied with significant disadvantage in the prognosis as compared to G-GIST. Furthermore, Kukar et al. found that younger patients with SI-GIST had a tendency to be presented with distant metastatic disease and larger tumor size [50]. In addition, the proportion of KIT exon 9 mutation was strikingly higher in SI-GIST than that in G-GIST, which may be the explanation of poorer prognosis of SI-GIST [52]. Inversely, several studies based on SEER database revealed comparable prognosis between small bowel and gastric GIST. After adjusting the confounding variables on a population based level, Guller et al. found that SI-GIST and G-GIST shared similar OS and CSS, which was contrary to common belief [17]. These results reflect those of Giuliano et al. [53] who further found that, although SI-GIST did have more aggressive features, SI-GIST patients were also more likely to undergo surgery than G-GIST (89.8% SI-GIST vs. 78.7% G-GIST), leading to the comparable survival outcomes. However, the previous studies always investigated SI-GIST as an entire cohort. As displayed in Fig. 2a, G-GIST seemed to share similar score with duodenal GIST (D-GIST), while jejunal GIST (J-GIST) patients were more likely to have LIM than ileal GIST (I-GIST) patients. Although I-GIST and J-GIST were reported to share compared prognosis in the study of Feng et al. [54], we suggested that more aggressive treatment should be taken into consideration to J-GIST for the high risk of LIM. In comparison with colon GIST (C-GIST), rectal GIST (R-GIST) tended to have more positive prognosis in spite of the less likelihood of surgical resection [50], analogous result could be found in the current study. Moreover, the aggressive course of extra-gastrointestinal GIST (EGIST) was suggested to be akin to SI-GIST [55], whereas according to the present LIM nomogram, EGIST seemed to shared similar prognosis with D-GIST, irrespective of I-GIST and J-GIST. Sample size and potential bias may result in such differences.

Unlike other solid tumors, lymph nodal involvement is extremely rare in GIST patients and lymph node dissection is not routinely suggested during the surgical treatment [56, 57]. However, in the present study, although the rate of lymph node metastasis (LNM) in the entire cohort was low (3.3%), LNM was significantly associated with LIM. This finding was consistent with that of Gaitanidis et al. who also found LNM was an independent prognostic factor of worse overall survival in patients with metastatic GIST [58], which further revealed that the evaluation of regional lymph nodes could be taken into consideration when undergoing surgical resection in patients with metastatic GIST. In addition, Li et al. found that lymphadenectomy was associated with an risk of mortality in GIST patients, which may be attributed to the destroy of the immune micro-environment in the normal lymph nodes and increasing postoperative morbidity and mortality caused by surgical trauma [59, 60]. What’s more, GIST patients with SDH complex deficiencies tended to have LNM. The SDH-deficient related disease like WT GIST (a component of the Carney-Stratakis syndrome) was reported to have high rates of LNM (29%) [61], hence the resection of enlarged nodes in SDH-deficient neoplasms was recommended in the National Comprehensive Cancer Network (NCCN) guidelines [62].

However, the current study is subject to several limitations. The study is a retrospective analysis, systematic and prospective data were lacked. External validation at other institutions was also lacked in our research, which may lead LIM nomogram to be overfitting. In addition, several critical clinicopathologic variables were required, especially the administration of tyrosine kinase inhibitors. If the information of co-morbidities, immunohistochemistry, and other laboratory values could be available for the construction of LIM nomogram, the results of our study might provide more valuable therapeutic measures for clinician.

## Conclusion

In conclusion, a large population-based cohort derived from the SEER dataset was screened for the construction of the novel nomogram for predicting LIM in patients with GIST. According to the results of the internal validation, DCA curve, and clinical impact plot, our nomogram could effectively predict the individualized risk of LIM. We hoped that the LIM nomogram could be further employed and improved in the clinical work, clinicians can choose better medical examinations and optimize therapeutic regimens with the help of LIM nomogram.

## Supplementary information

**Additional file 1: Table S1.** A database of the information of GIST patients obtained from SEER database.

**Additional file 2: Table S2.** A database of training set.

**Additional file 3: Table S3.** A database of testing set.

## Data Availability

The datasets used and/or analysed during the current study are available from the corresponding author on reasonable request.

## Abbreviations

SEER: Surveillance, Epidemiology, and End Results
GIST: Gastrointestinal stromal tumor
LIM: Liver metastasis
LNM: Lymph node metastasis
C-index: Concordance index
DCA: Decision curve analysis
OS: Overall survival
CSS: Cancer-specific survival
RFS: Recurrence-free survival
DFS: Disease-free survival
HPFs: High power fields
TKIs: Tyrosine kinase inhibitors
OR: Odds ratio
95% CI: 95% Confidence interval
ROC: Receiver operating characteristic
AUC: Area under ROC curve
